# Towards personalized prostate cancer screening

**DOI:** 10.1515/almed-2019-0027

**Published:** 2020-01-20

**Authors:** Xavier Filella

**Affiliations:** Department of Biochemistry and Molecular Genetics (CDB), IDIBAPS, Hospital Clínic, Barcelona, Catalonia, Spain

**Keywords:** prostate-specific antigen (PSA), screening, prostate cancer, PHI, 4Kscore

## Abstract

The value of the prostate-specific antigen (PSA) in prostate cancer (PCa) screening is controversial. Contradictory results have been reported in the literature as to whether PSA-based screening reduces mortality. Also, some of the studies published are methodologically flawed. However, evidence consistently demonstrates that screening programs results in the identification of patients with indolent prostatic tumors which rate has increased.

Controversy is not only about the value of PSA-based screening, but also about the age range for screening, risk groups based on baseline PSA, PSA ranges, or the use of other biomarkers (PHI, 4Kscore). At present, PCa screening in the general population is not recommended by most scientific societies, although it can be used after discussing the risks and benefits with the patient.

When discussing the need to perform a screening, the risks of using screening (lack of specificity of PSA, overdiagnosis) must be weighed against the risks of not performing it (increased rate of patients with initial diagnosis of metastasis). In the recent years, a number of authors have advocated the use of personalized screening, which could change the risk/benefit evaluation, thereby making screening necessary on the basis of a set of individual factors.

## Introduction

Prostate cancer (PCa) is the second most common type of cancer among men in the world and the most frequent in Europe [1]. Data has been recently published that it is also the most common type of tumor in Spain, with 33,370 new cases diagnosed in 2015 [2]. A rise has been observed in the incidence of PCa in Western countries in the last years, peaking in 2007. The predictive value of PSA in early diagnosis of PCa is controversial. Thus, PSA has a limited specificity and leads to overdiagnosis and overtreatment of numerous indolent, slow-growth tumors. As a result, clinical guidelines do not recommend the generalized use of PSA screening for PCa. Yet, PSA testing is not excluded, provided that the patient is informed of the risks and benefits of this practice.

## Screening studies

Published data on the usefulness of PSA screening for PCa have been provided in two large screening studies performed in Europe and USA: the European study titled *European Randomized Study of Screening for Prostate Cancer* (ERSPC), which included 162,387 men from seven European countries, and an American study titled *Prostate, lung, colorectal, and ovarian screening trial* (PLCO), which was performed in 76,693 men. The results obtained in the two studies were contradictory. According to the data published in 2011, the European study, with a mean follow-up of nine years, revealed a 20% reduction of mortality in the group that underwent PSA screening [3]. In contrast, with a follow-up of 7–10 years, the American study did not provide any evidence of PSA screening having any benefit [4]. In 2012, study updates with longer follow-up periods, confirmed the previous results obtained [5], [6]. The positive results of the European study were confirmed later with mean follow-up periods of 13 and 16 years [7], [8]. Conversely, negative results have been obtained in another recent study titled *Cluster Randomized Trial of PSA Testing for Prostate Cancer* as to the value of PSA screening [9]. This study, performed in UK, involved men of 50–69 years of age whose PSA levels were measured just once, as compared to the ERSPC and PLCO studies, where several measurements were made. Although the UK study included 415,357 men, it had some limitations such as the short follow-up time (only 10 years) and the low rate (40%) of biopsies performed when PSA test was positive.

On the other hand, the latest update of the European study reports that the number of subjects that need to be screened to prevent a PCa-death has progressively decreased as follow-up times have increased. Thus, the number needed to screen decreased from 1947 to 962, 742 and 570 after 9, 11, 13 and 16 follow-up years, respectively [8]. Consistently, the two latest updates of the European study support that the proportion of patients, diagnosed with metastasis or PSA above 100 μg/L was significantly higher in the control group, as compared to the group whose members underwent screening [7], [8].

Both, the European and American studies agree that the rate of diagnosis of PCa is higher in the group of patients who underwent PSA screening. This phenomenon results in overdiagnosis and overtreatment of a substantial number of tumors with a low risk of progression. Thus, based on data from the European study, the rate of low-risk tumors was 56.4% for the group who underwent screening versus 39.1% for the control group [8].

Also, the two studies [3], [4] consistently demonstrate that PCa-related mortality can only be reduced by PSA screening from 10 years of follow-up. The reason is that PCa progression is slow, with a broad window of treatment in most cases. Therefore, clinical guidelines do not recommend PSA testing in men with a life expectancy below 10 years.

Differences between the two studies [10] could be explained by methodological reasons. Thus, contamination by PSA testing in the control group was significantly higher in the American study (40–52%) as compared to the European study (15–20%), which would compromise the conclusions drawn in the PLCO study. Significant differences are also observed in the proportion of biopsies performed when screening was positive, namely, 86% in the European study versus 35% in the American study.

The heterogeneity of results across the seven sites of the European study is also worthy of note. Thus, positive results were obtained in the Sweden arm, where a 50% reduction was observed in mortality in the group of patients who underwent screening [11]. In contrast, the data published for the Spanish arm in the ERSPC trial does not show any differences in mortality based on the performance or not of screening tests [12]. Differences across ERSPC sites can be explained by variability in the periodicity of screening tests, rate of response to biopsy requests, level of contamination by PSA testing in the control group, or the treatments administered when a diagnosis of PCa was confirmed.

A recent microsimulation study revealed that the reduction of PCa-related mortality is associated with the type of protocol designed and adherence to it [13]. The authors observed that mortality increased when the model was designed based on ideal conditions, i. e., no contamination with PSA testing in controls, strict adherence to the protocol, or 100% response to biopsy requests. In this case, the study suggests that a 40% reduction could be achieved in PCa-related mortality by PSA testing.

## Reactions to screening studies

Some scientific associations have published clinical guidelines for PSA screening for PCa based on the results obtained in ERSPC and PLCO trials. Also, an influential review published by the *US Preventive Services Task Force* (USPSTF) in 2011, did not recommend PCa screening at all [14]. This review was based on six methodologically-robust studies, which showed that PCa screening results in minimal or nonexistent reduction in PCa-mortality. PCa screening was found to be associated with some prejudices against patient's examination and the administration of treatments that could be unnecessary. Based on the conclusions of this review, USPSTF [15] published, in 2012, its clinical guidelines where PSA screening for PCa was not recommended based on the poor risk/benefit balance of this practice.

A new period emerged, where recommendations became more conservative and advocated the reduction of PSA screening for PCa. In contrast, other authors support that PSA screening reduces the rate of patients with an initial diagnosis of metastatic PCa [7], [8], [16] and warn that failing to test PSA levels could result in increased rates of PCa-mortality [17]. Indeed, the USPSTF changed its point in 2018 and recommended that male patients 55–69 years of age should be offered PSA testing and informed on its associated risks and benefits [18].

## Efficacy of PSA in predicting the development of PCa in the long term

A range of studies support the efficacy of PSA in predicting the development of PCa years and even decades before it is diagnosed. This observation suggests that PSA release into the bloodstream is a risk factor for the development of PCa. This theory also involves that PSA have higher specificity in young than in older patients. The reason is that the rate of false positives is significantly higher in older patients as a result of the high prevalence of benign prostatic hyperplasia in men aged 60 years or older.

The first data on the predictive value of PSA in PCa was provided in a Finnish study published in 1994 by Stenman et al. [19]. In this study, 44 diagnoses of PCa were made in a cohort of 21,172 men aged 45–84 who were enrolled between 1968 and 1973. The authors concluded that a PSA >2.5 µg/L was predictive of the development of PCa. The following year, these conclusions were confirmed by Gann et al. [20] who increased the number of cases of PCa to 366. This study showed that, as compared to individuals with a PSA <1 µg/L, the relative risk of PCa and aggressive PCa increased as PSA concentrations were more elevated.

Other two larger studies based on longer follow-up periods supported the validity of PSA as a predictive marker of the development of PCa in the future. Loeb et al. [21], [22] reported that PSA levels in men younger than 60 years was a risk factor of developing PCa in the future. This study involved 26,000 men who were engaged in a PCa screening program between 1991 and 2001. The authors stated that the risk of receiving a diagnosis of PCa increased when PSA levels exceeded the median PSA levels for the general population, both for 40–49 year-old individuals (with a 14.6-fold increased risk) and for 50–59 year-old men (with a 7.6-fold increased risk).

Similar results were obtained by Lilja et al. [23] in a case-control study involving 21,277 men enrolled in a cardiovascular study between 1974 and 1986 to assess the predictive value of PSA. The authors measured PSA in 462 of the 498 men who were diagnosed with PCa, versus 1,222 matched controls, who were selected on the basis of their age and date of sampling. The results obtained revealed that PSA levels at 44–50 years of age were predictive of the risk of developing PCa in the future, which increased as PSA concentrations grew. Thus, the probability that a patient was diagnosed with PCa in the long term was 4% if PSA was <0.51 µg/L increased to 41% when PSA levels were 2.01–3 µg/L, and peaked to >60% when levels exceeded 3 µg/L.

More recent data were published by Vickers et al. [24], who demonstrated that PSA concentrations were not only predictive of a future diagnosis of PCa, but also were associated with the risk of developing metastatic PCa in the long term or die from this disease. This study involved 1,167 men of 60 years of age who underwent a blood sampling in 1981 and were monitored until 85 years of age. The study revealed that 90% of deaths from PCa occurred in men with a baseline PSA concentration >2 µg/L. These results are very relevant and facilitate the design of PCa screening programs. Baseline PSA concentration at 60 years of age would be predictive of the necessity of including or not a patient in a screening program. The benefits of screening would be higher in individuals with PSA >2 µg/L. Conversely, if PSA concentrations are <1 µg/L, the probability of developing PCa is low and, in the case a tumor was diagnosed, the probability of dying from it would be very low.

## PCa overdiagnosis and overtreatment

Overdiagnosis and overtreatment of PCa is a challenge to the development of an effective PCa screening program. Indeed, a high proportion of new diagnoses of PCa correspond to low-risk tumors. Based on ERSPC data, low-risk PCa account for 39.1% of diagnoses in the control group versus 56.4% in the screening group [8]. The risks and benefits of PSA screening for PCa are a matter of controversy. The reason is that a diagnosis of low-risk PCa will not benefit the patient, but can result in the patient undergoing an unnecessary radical prostatectomy or radiotherapy, which may cause urinary incontinence and erectile dysfunction, to name a few adverse effects.

Of special note are the results obtained in the PIVOT study (*Prostate Cancer Intervention versus Observation Trial*) published in 2017 after a 20-year follow-up [25]. The study included 731 men with localized cancer randomized to undergo either a radical prostatectomy or monitored by observation. The study demonstrated that surgery did not reduce PCa-related mortality and caused adverse effects more frequently. On the other hand, the study showed that surgery could reduce mortality in patients with intermediate risk of relapse, but not in patients with low-risk tumors.

The risks of overdiagnosis and overtreatment could be prevented if a protocol was designed to distinguish low-risk tumors from high-risk tumors. The active surveillance approach has been proposed as an effective tool for the management of patients with low-risk tumors that will never affect their life [26]. Patients with a Gleason grade <7, PSA concentration <10 µg/L and a low proportion of tissue infiltrated by the tumor in the biopsy can be included in active surveillance programs. These programs involve monitoring patients by an active follow-up, and delay the administration of an active therapy until tumor progression. Thus, PSA measurements, rectal exams and regular biopsies to assess whether Gleason score increases should be performed regularly.

The correct classification of tumors is key to distinguish patients who need radical surgery from those with low-risk PCa who can be monitored by active surveillance. Yet, the variables currently used for patient selection are a matter of controversy. On the one hand, Gleason score as calculated from a biopsy is not 100% effective as a result of tumor heterogeneity and sampling inaccuracy. On the other hand, PSA levels can be measured using a range of techniques that yield different results. Yet, clinical guidelines recommend that patients are selected based on a 10 µg/L cut-off point, regardless of the PSA measurement system employed [27]. In addition, PSA levels may be very high in PCa patients with a large prostate, which are not related to tumor aggressiveness but to prostate volume. As a result, based on their PSA levels, these patients may be excluded from active surveillance protocols. Some authors suggest that PSA density could be more useful than PSA levels in the selection of patients for active surveillance [28]. Nevertheless, positive results have been obtained in active surveillance protocols, with 70% of patients untreated after five-year follow-up and a risk of PCa-related mortality at 15 years of only 3% [29].

## Clinical guidelines

PCa screening is, even today, a controversial question. Prior to a PCa screening, its associated risks and benefits should be weighed on a case-by-case basis. However, clinical guidelines occasionally provide contradictory recommendations. Table 1 contains a summary of recommendations from five relevant scientific associations [30], [31], [32], [33], [34] which, despite some differences, agree that patients should be adequately informed and involved in the decision-making process.

**Table 1:** Summary of clinical guidelines for PCa screening.

Clinical guidelines, year (reference)	General recommendation	Additional information	Other recommended biomarkers
EAU–ESTRO–SIOG, 2017 [30]	Offer an individualized risk-benefit weighing to well-informed men with a life expectancy of at least 10–15 years.	Offer PSA assessment to men older than 50, older than 45 with family history of PCa, Afro-American older than 45, men with PSA >1 µg/L at 40 years or >2 µg/L at 60 years.	Free PSA enables PCa risk stratification in men with a PSA of 4–10 µg/L and a previous negative biopsy. New risk stratification assays that include PHI and 4Kscore to spare men with a PSA of 2–10 µg/L unnecessary biopsies.
AUA, 2018 [31]	For 55–69 year-old men, weigh the risks and benefits associated with screening and active therapies. Screening is not recommended in men younger than 54 years with an intermediate risk of PCa, in men older than 70, or in men with a life expectancy below 10–15 years.	Decision should be made on a case-by-case basis for men younger than 55 years with a high risk of developing PCa, i. e., Afro-American and men with a family history of metastatic cancer.	PSA derivatives (PSA density, specific ranges according to the age of the patient), PSA kinetics (velocity and doubling time), percentage of free PSA, proPSA and PCA3 should be considered secondary tests potentially useful to determine the need to perform or repeat a prostate biopsy.
USPSTF, 2018 [32]	The decision should individualized for 55–69 year-old men. The potential risks and benefits of screening should be previously discussed with the patient. Screening should not be performed in men older than 70 years.	Screening should not be performed in individuals younger than 55 years with an intermediate risk of PCa nor even to obtain baseline PSA. Men with a family history of PCa and Afro-Americans are at a high risk of developing PCa.	Not recommended
NCCN, 2019 [33]	The decision to participate in an early PCa screening should be made after an adequate weighing of its associated risks and benefits. There is no general agreement as to the age range at which PCa screening should be performed.	Most experts agree that screening should start at 45 years. Screening will be repeated at 2–4-year intervals in men 45–75 years of age if PSA is <1 µg/L, or at 1–2-year intervals if PSA is >1 µg/L.	A percentage of free PSA <10%, PHI >35 or 4Kscore (which shows the probability of developing a high-risk PCa) are potential indicators of the need to perform a biopsy. A PCA3 >35 is potentially informative after a previous negative biopsy.
NICE, 2019 [34]	The decision to obtain a prostate biopsy should not be made only on the basis of PSA concentration. A MRI scan should be the first-choice test for individuals with clinical symptoms of localized PCa. Adequately-informed patients have the right to participate in decision-making.	Family history, and PSA density and velocity should be assessed when considering a biopsy in individuals with a low risk of developing PCa based on MRI results, and high PSA concentrations.	PCA3 and PHI are not recommended for individuals with clinical symptoms of PCa whose biopsy was negative.

PCa, prostate cancer; *PCA3*, Prostate Cancer 3 gene; PHI, Prostate Health Index; PSA, Prostate-specific antigen; AUA, American Urological Association; EAU, European Association of Urology; ESRO, European Society for Radiotherapy and Oncology; NCCN, National Comprehensive Cancer Network; NICE, National Institute for Health and Care Excellence; SIOG, International Society of Geriatric Oncology; USPSTF, US Preventive Services Task Force.

There is no general agreement on the age range at which screening should be offered. According to the American Urological Association (AUA) and the USPTF, screening should be considered in 55–69 year-old patients at an average risk of developing PCa. In contrast, the guidelines collaboratively designed by the European Association of Urology (EAU), the European Society for Radiotherapy and Oncology (ESRO) and the International Society of Geriatric Oncology (SIOG) recommend that screening should be offered to all men older than 50 with a life expectancy of 10–15 years, and to all young men who exceed a specific PSA cut-off point. Although the National Comprehensive Cancer Network (NCCN) acknowledges that there is no agreement among the authors of the guidelines, they recommend that screening is offered to men from 45 years of age. In addition, NCCN guidelines recommend that PSA concentrations should be considered when deciding the periodicity with which PSA tests should be performed. The NCCN does not establish a specific age from which screening should not be performed, whereas other guidelines (EAU–ESTRO–SIOG, AUA and USPSTF) do not recommend screening from 70 years of age in individuals with a life expectancy below 10–15 years.

Finally, the National Institute for Health and Care Excellence (NICE) guidelines do not consider screening and recommend that a MRI should be performed in patients with symptoms suggestive of localized PCa. In addition, NICE guidelines recommend measuring PSA density and velocity in patients at low risk of PCa based on MRI results.

New biomarkers as the PCA3 score, prostate health index (PHI) or proPSA and 4Kscore, are mentioned in EAU–ESTRO–SIOG, AUA and NCCN guidelines as potentially useful. Conversely, they are not recommended in USPTF or NICE guidelines.

## Conclusions

**Figure 1:**
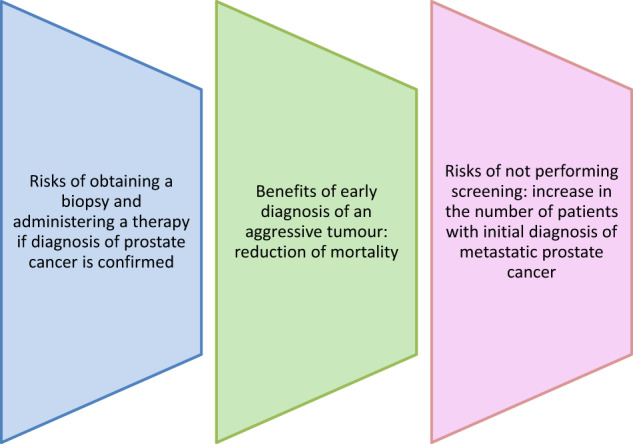
Risks and benefits of early detection of prostate cancer.

PCa is a highly challenging disease, with significant prognostic differences that range from indolent tumors that will never affect patients, to lethal tumors resistant to castration. This heterogeneity influences decisively the risk/benefit balance of screening (Figure 1). Early diagnosis of a tumor will only benefit those patients with aggressive tumors for which effective therapies that reduce cancer-related mortality are available. On the other hand, screening involves the risks associated with biopsy and therapies in case PCa is detected. Additionally, a large number of negative biopsies will be obtained as a result of the low specificity of PSA. Furthermore, the risks associated to treatment have special relevance, as a high proportion of PCa patients will have low-risk tumors that do not require any therapy. Active surveillance protocols spare patients the negative effects of an active therapy that is unnecessary in indolent tumors.

**Figure 2:**
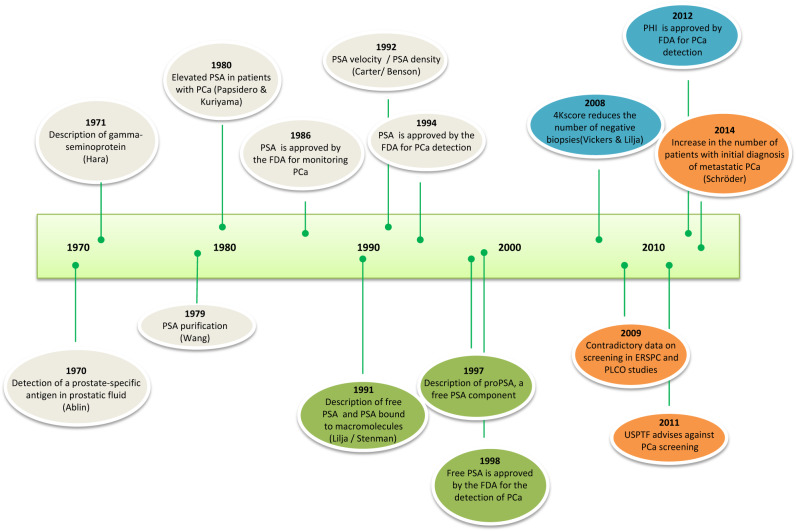
Timeline of biomarkers used for the detection of PCa.

The identification of new biomarkers that are more specific than PSA and are related to tumor aggressiveness can help improve the results of screening programs, thereby reducing their associated risks. In the '90, PSA was known to be a molecule made up of a free fraction and other fractions bound to a set of macromolecules, primarily to alpha-1-antichymotrypsin. Many other fractions have been identified in the last decades, such as intact PSA, BPSA and proPSA (Figure 2). The identification of these new PSA fractions has enabled the development of new biomarkers, including PHI and 4Kscore. According to published data, these tests are more specific than PSA and related to tumor aggressiveness, thereby facilitating risk stratification. These tests will be useful to distinguish patients with indolent PCa from those with aggressive prostate tumors, and establish the necessity of administering a therapy or not.

PHI, which combines total PSA, free PSA and -2 fraction of the proPSA (p2PSA) from the formula (p2PSA/free PSA)*total √PSA, has higher effectiveness than PSA and the percentage of free PSA in the detection of PCa, with areas under the curve ranging from 0.67 to 0.781 according to the literature [35]. In June 2012, the *Food and Drug Administration* (FDA) approved the use of this test to decide whether a biopsy is warranted in men older than 50, a PSA of 4–10 µg/L, and a negative rectal exam. Based on our experience, PHI is related to prostate volume, with areas under the curve of 0.818, 0.716 and 0.654 for patients with prostate volumes of ≤35, 36–50 and >50 cc, respectively [36].

4Kscore is an index calculated from total PSA, free PSA, intact PSA and human kallikrein 2, along with the age of the patient, rectal exam and presence or not of a previous negative biopsy. The aim of this test – also known as the 4-Kallikrein test – is not PCa detection, but the detection of high-risk PCa. The results of a series of retrospective studies with this test demonstrate a substantial improvement with respect to the use of PSA only, with areas under the curve of 0.798 and 0.903, respectively [35]. These results are supported by a recent study, with an area under the curve of 0.821 [37].

The studies performed on the performance of these tests show that the cost-effectiveness of early diagnosis of PCa improve with the use of PHI [38], [39] and 4Kscore tests [40]. Some scientific associations recommend the use of these tests, although there is no general agreement on their use.

The risks of not performing screening should be considered when weighing the risks and benefits of PCa screening. In fact, numerous studies show an increase in the rate of patients with an initial diagnosis of metastatic PCa when screening was not performed [8], [16], resulting in higher rates of PCa-related mortality [17].

The usefulness of PSA screening for PCa is a matter of controversy, which is manifested in the contradictory recommendations made by the different scientific organizations [41]. Controversy is not limited to the uncertainty associated with screening and includes the age range in which screening should be performed, the definition of risk groups based on baseline PSA, the periodicity of PSA measurements or the usefulness of other biomarkers that improve PSA effectiveness. We performed a critical review of studies on PSA screening published to date – some of which were methodologically flawed – and support the results of some of these studies [42]. We support that the risks and benefits of PSA screening for PCa should be weighed before a decision is made on the need to perform a PSA screening test or not. When weighing the risks associated with PSA screening, the risks associated with failing to perform PSA screening should also be considered. In the last years, new data support that the decision to make or not a PSA screening should be made on a personalized way. Eapen et al. [43] support an individualized decision-making, as opposed to the dichotomy between a general population screening protocol and a not-screening anyone protocol. In the same vein, Carlsson and Roobol [44] advocate a new-generation screening protocol based on new biomarkers and risk stratification based on baseline PSA. Recommendations of personalized screening introduce a new paradigm for reflection that could change the risk/benefit balance of PCa screening based on the risk of each patient.
